# Perinatal mental health among women in Somalia a silent emergency demanding urgent health system action

**DOI:** 10.3389/fgwh.2026.1859985

**Published:** 2026-08-06

**Authors:** Sharmake Gaiye Bashir, Hiba Abdi Salad, Yakub Burhan Abdullahi, Yusuf Hared Abdi, Mohamed Sharif Abdi, Naima Ibrahim Ahmed

**Affiliations:** Department of Public Health, Faculty of Medicine and Health Science, Hormuud University, Mogadishu, Somalia

**Keywords:** emergency, fragile health systems, maternal health, perinatal mental health, Somalia

## Abstract

Perinatal mental health among women in Somalia represents a critical yet neglected public health emergency shaped by three decades of protracted conflict, gender-based violence, internal displacement, and one of the world's weakest health systems. With a maternal mortality ratio of 621 per 100,000 live births and fewer than 21% of births occurring in health facilities, depression and anxiety affect approximately one in four perinatal women—yet remain entirely absent from routine maternal care. This Perspective examines the structural, policy, and systemic barriers that perpetuate this neglect and proposes evidence-informed pathways for urgent action: integration of screening and brief interventions into primary healthcare, task-shifting to community health workers, culturally adapted psychosocial interventions, and strengthened governance frameworks. We emphasize that addressing perinatal mental health is foundational to Somalia's health system recovery, critical for reducing maternal and neonatal mortality, and essential for safeguarding the development and wellbeing of a generation of Somali children.

## Introduction

Perinatal mental health conditions are among the most common complications of pregnancy and childbirth globally, with pooled prevalence of perinatal depression and anxiety approaching one in three women in low- and middle-income countries (LMICs) (1, 2). In fragile and conflict-affected settings, these burdens are amplified yet remain largely invisible within health policy and service delivery. Somalia, with documented conflict-related trauma and minimal mental health infrastructure, illustrates this “silent emergency.” However, precise prevalence data specific to Somalia remain unavailable. where perinatal mental health is critical to both maternal survival and long-term child development but almost entirely absent from routine care (3, 4). As a Perspective, this article synthesizes published evidence from LMICs, sub-Saharan African, and Somali-region literature to build a policy-relevant argument for health system action, rather than presenting primary data or a systematic review.

Somalia's three-decade protracted conflict, recurrent droughts, displacement, and entrenched gender inequality create an extreme risk environment for perinatal mental disorders. While maternal and newborn health have gained some prominence in global and national agendas, perinatal mental health has not, despite strong evidence linking untreated perinatal mental disorders to adverse maternal and child outcomes. Addressing perinatal mental health in Somalia is therefore framed here as a neglected public health emergency demanding urgent, system-wide action and as foundational to achieving universal health coverage and the Sustainable Development Goals in fragile settings.

This Perspective is organized around three intersecting dimensions: the epidemiological burden of perinatal mental disorders in the Somali context; the structural, systemic, and sociocultural barriers that perpetuate neglect; and evidence-informed pathways for health system action.

## Burden and context in Somalia

Somalia's population has been exposed to cumulative trauma, displacement, and chronic poverty. A WHO study cited in a recent national review indicates Somalia has among the highest mental illness rates globally, with widespread psychosocial trauma, social deprivation, and substance use (3). Women bear a disproportionate share of this burden due to gender-based violence, restricted decision-making power, and economic dependence (3, 4). Evidence from the Somali Region of Ethiopia a geographically proximate but distinct population and broader African settings suggest antenatal depression may affect around one in four women and is associated with low social support, chronic illness, and prior depression (4, 5).

African data show that women with perinatal mental disorders have markedly higher risks of preterm birth, low birthweight, neonatal complications, non-exclusive breastfeeding, and infant mortality (4, 6). Among refugees and forced migrant populations highly relevant to Somalia pooled prevalence of perinatal depression exceeds 30%, with post-traumatic stress disorder (PTSD) reaching 17% in forced migrants (7, 8). These patterns from neighboring regions and refugee populations provide indirect evidence but cannot be directly extrapolated to Somalia. The absence of local epidemiological data means estimates remain uncertain and contingent on proxy populations. Somali women's mental health burden likely differs due to distinct conflict patterns, health system structures, and cultural contexts.

## Health system gaps and structural barriers

Somalia's mental health system is profoundly under-resourced: mental health beds in general hospitals are approximately 0.5 per 100,000 population, a fraction of regional and global averages, and the specialist workforce is extremely limited (3). Mental health services remain largely centralized, urban, and hospital-based, with minimal integration into primary healthcare (PHC) or maternal, newborn, and child health (MNCH) platforms (3, 6). In East Africa, qualitative evidence highlights poor quality care, provider shortages, inadequate supplies, abusive behaviors, and high costs as major barriers to maternal healthcare access (9). These systemic weaknesses are magnified in Somalia's conflict-affected context, where insecurity, damaged infrastructure, and limited transport further constrain service use (3).

Financing constraints are severe: mental health receives minimal domestic allocation and is heavily dependent on fragmented external funding (3, 10). Out-of-pocket payments are common, creating catastrophic cost risks for families seeking specialist care (3). Stigma, fear of being labeled “mad,” and strong reliance on traditional and religious healers shape help-seeking, often delaying or diverting women from evidence-based care (3, 4). For perinatal women, intersecting barriers limited autonomy, male-controlled finances, and social norms that trivialize emotional distress further restrict access to both maternal and mental health services (4).

## Why perinatal mental health remains neglected

Policy neglect is central. Systematic mapping of mental health research and policy in Somalia shows dominance of generic service and policy debates, with little focus on women's mental health consequences or perinatal conditions as a distinct priority area (3). At continental level, maternal mental health remains weakly integrated into policies, under-funded, and rarely translated into programmatic responses (4).

Donor-driven programming in Somalia has prioritized emergency stabilization, communicable diseases, and nutrition, often through vertical projects. This architecture fragments service delivery, with short-term, projectized psychosocial interventions disconnected from routine MNCH and PHC systems (3, 11). Perinatal mental health falls into a gap between mental health, protection, and reproductive health silos, leading to duplication in some areas and absence in others.

Within maternal health programs, an overwhelming focus on reducing mortality and addressing obstetric emergencies leaves little room for non-communicable, non-fatal conditions such as depression, anxiety, or PTSD (3, 6, 9). Screening for common perinatal mental disorders is rarely institutionalized, despite high-quality evidence that structured screening programs can significantly reduce depressive and anxiety symptoms when linked to care (3). As a result, perinatal mental disorders remain “invisible morbidity,” affecting outcomes yet not measured, financed, or managed.

## Pathways for urgent health system action

Somalia's move toward universal health coverage and PHC revitalization offers a critical window to embed perinatal mental health into core service packages. Global LMIC evidence indicates that integration of mental health into perinatal care, using stepped-care models and WHO's mhGAP guidance, is both feasible and cost-effective in relatively stable settings (6, 12); however, Somalia's low ANC attendance rates, particularly in rural and conflict-affected areas, mean that antenatal contact alone cannot be assumed as a reliable entry point for screening and intervention. Somalia's ANC attendance is 27% nationally; 50% of women deliver outside facilities (13). Perinatal screening cannot rely on antenatal contact. Alternative entry points include: postnatal home visits by CHWs (feasible in settled areas), community immunization clinics, nutrition programs, and IDP camp health posts. Each requires distinct training, logistics, and funding but reaches women who bypass formal ANC. IDP camps currently employ basic health workers; mental health training requires collaboration with humanitarian partners and security agreements. In such models, routine antenatal and postnatal visits become entry points for psychosocial assessment, brief interventions, and referral. Implementing simple, validated tools for depression and anxiety screening adapted linguistically and culturally within ANC, delivery, and postnatal contacts can identify high-risk women, provided that pathways to care are in place.

Task-shifting is essential in Somalia's context of extreme workforce scarcity. Evidence from LMICs shows that non-specialist providers and community health workers (CHWs) can safely deliver psychoeducation, basic counseling, and structured psychosocial interventions, with outcomes comparable to physician-led care and potential cost savings (12, 14). Frameworks such as the C4 model delineate roles across lay workers, community-level counselors, PHC providers, and specialists, enabling scalable, community-based mental health services even in low-resource, conflict settings (15). In Somalia, task-shifting barriers are severe. (1) **Workforce retention:** CHWs face irregular pay and security threats; salaries must be guaranteed through government or sustained donor commitment. (2) **Supervision gaps:** Clinical supervision infrastructure does not exist; remote supervision via phone requires mobile network expansion. (3) **Drug supply:** Psychotropic medicines are unavailable in most facilities; procurement pathways must be established first. (4) **Geographic coverage:** Insecurity limits access to rural and herder populations. These must be solved before training alone; phased pilots in secure urban/peri-urban areas should precede rural rollout.

Cultural adaptation is non-negotiable. Evidence from African and Somali-region settings underscores the role of social adversity, marital conflict, and limited support as key determinants of perinatal depression (2, 4, 5). Interventions such as the Thinking Healthy Programme and other cognitive-behavioral approaches have been adapted for delivery by CHWs, emphasizing mother-infant bonding and family engagement (6). In Somalia, co-design with women, families, religious leaders, and traditional birth attendants is critical to ensure that concepts of distress, idioms of mental illness, and healing practices are respected while challenging harmful norms and stigma (3).

Digital health offers opportunities but also risks. Across Africa, digital maternal and child health (MCH) interventions SMS, apps, and wearable devices have expanded rapidly, but maternal mental health remains severely under-represented, and many interventions overlook local family structures and low literacy (11, 16). Systematic reviews highlight that digital mental health tools are often perceived as impersonal, can increase provider workload, and face regulatory and equity barriers (11, 16). In Somalia, where connectivity is uneven and women's phone access is gendered, digital solutions should be viewed as adjuncts rather than substitutes: for example, supporting supervision of CHWs, remote training, or simple voice-based follow-up, rather than stand-alone apps for self-management. Policy frameworks must guard against digital innovations exacerbating inequalities by privileging urban, literate, or wealthier women.

Policy and governance reforms are foundational. Somalia can leverage WHO's Special Initiative for Mental Health and global guidance on integrating perinatal mental health into MNCH to articulate a clear national strategy, with explicit inclusion of perinatal mental health indicators within health information systems and performance frameworks. Aligning donor investments with a national perinatal mental health plan rather than isolated pilots would reduce fragmentation and enable sustainable financing for training, supervision, psychotropic medicines, and community-based psychosocial programs. Given Somalia's fragmented governance (federal, regional, state, municipal actors), competing health priorities, and 90% health financing dependency on donors, implementation must be phased: Phase 1 (Years 1-2): Pilot screening and brief counseling in 3 secure urban health facilities; build supervision capacity. Phase 2 (Years 3-4): Expand to 15 facilities; integrate into health information systems. Phase 3 (Years 5+): National scale with domestic budget allocation. Parallel advocacy must secure WHO endorsement and align international donors (World Bank, Global Fund) to coordinate financing.

Embedding perinatal mental health within broader efforts to address health inequalities in post-conflict settings through social protection, gender-responsive policies, and legal protections against gender-based violence will be essential to tackle upstream determinants.

## Conclusion

Perinatal mental health in Somalia is a silent emergency at the intersection of protracted conflict, gender inequity, and health system fragility. Ignoring it undermines maternal survival, newborn outcomes, and the prospects of a generation of Somali children. Evidence from Africa and other LMICs demonstrates that integrating perinatal mental health into PHC and maternal services, backed by task-shifting, culturally grounded psychosocial interventions, and prudent use of digital tools, is both feasible and impactful.

For Somalia and its partners, positioning perinatal mental health as an essential component of maternal health is not an optional add-on but a core strategy for rebuilding a resilient, people-centred health system. Immediate policy commitments, aligned financing, and sustained implementation are required to ensure that every pregnant and postpartum woman whether in Mogadishu, a rural village, or an IDP camp has access to compassionate, competent mental healthcare as part of routine care. Addressing this silent emergency is integral to advancing universal health coverage, gender equity, and sustainable peace.

## Data Availability

The original contributions presented in the study are included in the article/Supplementary Material, further inquiries can be directed to the corresponding author/s.
